# The Korean Study of Women’s Health-Related Issues (K-Stori): Rationale and Study Design

**DOI:** 10.1186/s12889-017-4531-1

**Published:** 2017-06-29

**Authors:** Ha Na Cho, Eunji Choi, Da Hea Seo, Mina Suh, Hoo-Yeon Lee, Boyoung Park, Sohee Park, Juhee Cho, Sue Kim, Yeong-Ran Park, Joong-Yeon Lim, Younjihin Ahn, Hyun-Young Park, Kui Son Choi, Yumie Rhee

**Affiliations:** 1 Graduate School of Cancer Science and Policy, National Cancer Center, 323, Ilsan-ro, Ilsandong-gu, Goyang-si, Gyeonggi-do 10408 Republic of Korea; 2 Department of Endocrinology and Metabolism, Inha University School of Medicine, Incheon, Republic of Korea; 3 National Cancer Control Institute, National Cancer Center, Goyang, Republic of Korea; 4 Department of Social Medicine, College of Medicine, Dankook University, Cheonan, Republic of Korea; 5 Graduate School of Public Health, Yonsei University, Seoul, Republic of Korea; 6 Department of Clinical Research and Evaluation, Sungkyunkwan University, Seoul, Republic of Korea; 7 College of Nursing, Yonsei University, Seoul, Republic of Korea; 8 Division of Silver Industry, Kangnam University, Yongin, Republic of Korea; 9 Division of Cardiovascular Diseases, Center for Biomedical Science, Korea National Institute of Health, Osong, Republic of Korea; 10 Department of Internal Medicine, Endocrine Research Institute, Severance Hospital, Yonsei University College of Medicine, Seoul, 03722 Republic of Korea

**Keywords:** Women’s health, Life cycle, Adolescence, Childbearing, Pregnancy, Postpartum, Menopause, Elderly

## Abstract

**Background:**

Measures to address gender-specific health issues are essential due to fundamental, biological differences between the sexes. Studies have increasingly stressed the importance of customizing approaches directed at women’s health issues according to stages in the female life cycle. In Korea, however, gender-specific studies on issues affecting Korean women in relation to stages in their life cycle are lacking. Accordingly, the Korean Study of Women’s Health-Related Issues (K-Stori) was designed to investigate life cycle-specific health issues among women, covering health status, awareness, and risk perceptions.

**Methods:**

K-Stori was conducted as a nationwide cross-sectional survey targeting Korean women aged 14–79 years. Per each stage in the female life cycle (adolescence, childbearing age, pregnancy & postpartum, menopause, and older adult stage), 3000 women (total 15,000) were recruited by stratified multistage random sampling for geographic area based on the 2010 Resident Registration Population in Korea. Specialized questionnaires per each stage (total of five) were developed in consultation with multidisciplinary experts and by reflecting upon current interests into health among the general population of women in Korea. This survey was conducted from April 1 to June 31, 2016, at which time investigators from a professional research agency went door-to-door to recruit residents and conducted in-person interviews.

**Discussion:**

The study’s findings may help with elucidating health issues and unmet needs specific to each stage in the life cycle of Korean women that have yet to be identified in present surveys.

## Background

Women’s health care throughout the world has transformed to include issues related to various social and environmental determinants of women’s health [1]. The strongest international statement on women’s health emerged from the United Nations Fourth World Conference on Women held in September 1995 in Beijing. The resulting Platform for Action addressed the following topics: Improving access to appropriate, affordable, and quality health care for women in all stages of the female life cycle, as well as information and related services, strengthening prevention services to promote women’s health, and ensuring reproductive health rights. Also, a clear commitment was made to securing resources with which to manage and monitor women’s health continuously [2]. Since the 1995 Beijing Declaration, Korea has moved to expand support for women’s health by implementing policies and developing infrastructure to ensure the right to health among women. Meanwhile, however, routine health surveys to monitor and identify women’s health issues according to individual stages of the female life cycle have not been conducted. As presently ongoing, national health surveys in Korea are not designed to identify health issues specific to women, the data therefrom limit researchers’ ability to fully elucidate life cycle problems facing women. Also, since current questionnaires were developed without understanding gender influences and social contexts, monitoring unmet health needs over the life cycle of women is difficult [3–5].

In Korea, the female population accounts for 50% of the total population, and women older than 60 years outnumber their male counterparts [6]. Previously, Women’s Health Statistics and Facts in Korea reported that Korean women have a higher life expectancy than Korean men, despite expressing lower levels of self-rated health, indicating a lengthy period of unhealthiness among women of older age [7]. Meanwhile, a lack of policies specific to protecting women’s health, in addition to economic, political, and social vulnerability, impose various health problems on Korean women [7]. In order to implement successful policies through which to improve women’s health, regular surveillance thereof is essential. Accordingly, the Korean Study of Women’s Health-Related Issues (K-Stori) was initiated to investigate health statuses and health perceptions among Korean women according to stages in the female life cycle and to identify health issues in each stage thereof. The aim of this article was to describe the rationale for and the design of the survey in the K-Stori.

## Methods/design

### Study design and subjects

The K-Stori is a nationwide survey designed to investigate broad health issues among Korean women according to five stages in the life cycle of women. Specific questionnaires were designed for each of the five stages (adolescence, childbearing, pregnancy & post-partum, menopause, and older adult), since life cycle approaches have been found to be effective in understanding and managing health problems and health promotion plans. In total, 15,000 women were recruited (3000 women per stage). Eligibility for inclusion and survey methods for each life cycle stage are shown in Table 1. The target population comprised women between 14 and 79 years of age for all households in Korea. Institutional households were excluded. Subjects who did not agree to participate in the survey and those with difficulties communicating were excluded.

**Table 1:** Eligibility and survey method for each life cycle stage

Women’s life cycle	Age	Sample size	Survey method	Inclusion criteria
Adolescent girls	14–17	3000	Online survey	Middle school 2nd grade and high school 2nd grade
Childbearing women	19–44	3000	Household interview	Not pregnant or no childbirth within 1 year
Pregnant women	19–44	3000	Interview at obstetrics and postnatal center	During pregnancy or birth experience within a year
Menopausal women	45–64	3000	Household interview	Menopausal transition period or no menstruation within a year
Older adult women	65–79	3000	Household interview	

**Fig. 1:**
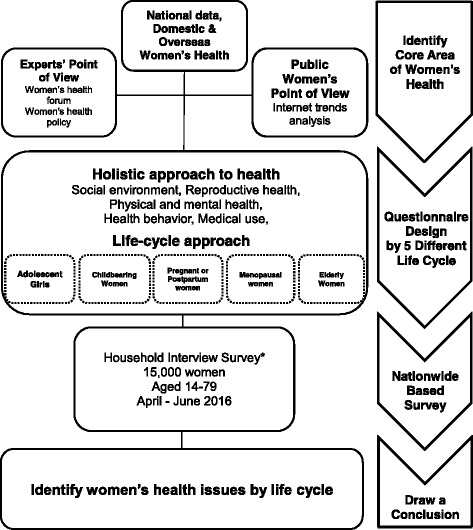
Research Framework for K-Stori. * Online survey was conducted for adolescent, and interview was conducted at obstetrics and postnatal centers for pregnant women

Considering the generalization of survey results, in-person interviews were carried out. However, for adolescents, the questionnaire was conducted via an online survey to minimize distortion of responses to sensitive health behavior items (Fig. 1).

### Sampling

We randomly sampled 3000 women in each stage of the life cycle for a reliable and representative research design. Weights are presented to compare data among stages. For random sampling, a multi-level, stratified, probability-proportional statistics extraction method was used as a sampling framework using the 2010 Population and Housing Census. In order to generalize the survey results, the subjects were selected by random sampling for 16 cities and provinces (seven special and metropolitan cities, and nine provinces). The extraction method was used to stratify recruits by region and dong-eup/municipal district, after which 200 households were sampled. A sample of 15 households was extracted from each sampling area, in which the principle was to survey female household members aged 14–79 years in each sample household. Interviewers visited each of the 15 extracted households.

To recruit women in adolescence and pregnancy/post-partum period, interviewers planned to visit schools and obstetrics and gynecology or post-partum care centers. In order to select the same survey area as for the other life cycle stages, a system extraction method was used to identify local schools for adolescents and obstetrician and post-partum care centers for pregnant and post-partum women based on the same sample design area for the household survey subjects. If there were no schools or obstetrics and gynecology clinics in the sampling area, they were replaced by the nearest one.

### Sample size calculations

Statistical power according to various odds ratios was calculated in order to examine whether the sample number of 3000 women for each life cycle stage was sufficient. Logistic regression analysis was used to calculate the statistical power that can be detected by various odds ratios when the risk group was assumed to be approximately 20% of the total subjects and when the outcome prevalence of the unexposed group was assumed to be about 10%. With these assumptions, the statistical power that can detect an OR = 1.6 was about 82% for surveying 3000 women in each life cycle stage. In addition, for subgroup analysis of a particular group of subjects (e.g., 1000 to 2000 persons), sufficient statistical power will be obtained in most analyses.

Assuming a 20% exposure to risk factors and a prevalence of 10% in the unexposed group is conservative. For general analysis, the analysis will be divided for 50% of the exposed and unexposed groups. As the prevalence of the outcome variable increases, the statistical power will be much higher. Therefore, the number of subjects to be surveyed in this study is expected to have sufficient power for statistical analysis of the survey data on women’s health issues.

### Design of survey questionnaires

The survey questionnaires were structured differently for each life cycle stage to identify stage-specific health issues. Three strategies were implemented to develop a questionnaire. First, we analyzed national health data to understand the state of women’s health in Korea. We also reviewed both domestic and foreign health behavior-related surveys, especially those developed for women. Second, questionnaires were developed by gathering opinions from a number of experts: The Women’s Health Forum was held to diagnose women’s health problems, and multidisciplinary experts directly participated in the questionnaire development. Experts in each field identified issues related to women’s health from their viewpoint. The fields covered by these experts included medicine, public health, preventive medicine, statistics, nursing, geriatrics, communication health, social welfare, and sociology. Third, we analyzed health issues of interest to women through internet information analysis and focus group interviews. Using keywords, such as “women, health,” we searched the web and mapped blogs and internet cafes most used by women in Korea during January to October 2015. Focus group interviews were also conducted to identify women’s health issues and unmet needs in health care. Through these processes, we identified areas to be investigated for each life cycle stage. In questionnaire development, we tried to adopt questionnaire items that have been validated in previous studies.

The questionnaire items and the assessment tools adapted in this survey are shown in Table 2. The main questionnaire items assess health status, perceived health, reproductive health, physique and body type, physical activity and exercise, eating habits, food intake, smoking, drinking, sleeping, medical service use, health communication, social support and relationships, violence, social-economic status, and demographic characteristics. Each of the five stage-specific questionnaires consists of 26 questions common among all questionnaires and more than 100 questions specified to each life cycle stage.

**Table 2:** Questionnaires items and assessment tools in K-Stori

Survey area	Survey Tool or Main Content	Adolescent girls	Childbearing women	Pregnant /Post-partum women	Menopausal women	Older adult women
A. Health status	-Self-reported health status, Happiness, Quality of Life [3]	O	O	O	O	O
	-Rosenberg Self-esteem scale [8, 9]	O	O	O	O	O
	-Perceived Stress Scale 4 (PSS4)^a^ [10, 11]	O	O	O	O	O
	-Parenting Stress [12]			O		
	-Patient Health Questionnaire 9 (PHQ9)^b^ [13, 14]	O	O		O	
	-Edinburgh Postnatal Depression Scale (EPDS)^c^ [15, 16]			O		
	-Geriatric Depression Scale (GDS)^d^ [17]					O
	-Suicide	O	O	O	O	O
	-Incontinence, Fall, Activity in Daily Living					O
AA. Perceived health	-Perceived health risk, risk factors	O	O	O	O	O
	-Perceived health threat	O	O	O	O	O
B. Reproductive health	-Medical Outcomes Study Questionnaire Short Form 36 Health Survey (SF36)^e^ [18]	O	O			
	-Premenstrual syndrome (PMS)^f^ [19]	O	O			
BB. Menopause	-Menopause-related questionnaire [20]				O	
C. Body Shape	-Satisfaction and ideal body image, Stunkard Figure Rating Scale [21]	O	O		O	O
	-Trying to lose weight [4]	O	O		O	O
	-Social norms on plastic surgery and beauty treatment behavior [22]		O		O	O
D. Physical Activity	-International Physical Activity Questionnaire (IPAQ)^g^ [23]		O		O	O
E. Eating Habits	-Average meal a day, Dine out [4]	O	O		O	O
	-Bulimia and Anorexia Nervosa [24]	O	O			
F. Food Intake	-Supplement intake [4]		O	O	O	O
	-Dairy food, Coffee, Soda and Highly Caffeinated Drinks intake [25]	O	O		O	O
G. Smoking	-Smoking history, Exposure of Second hand smoking [4]	O	O	O	O	O
H. Drinking	-Social norms for drinking [22, 26]	O				
	-Drinking experience [4]	O	O	O	O	O
	-Tolerance Annoyance Cut down Eye opener (T-ACE Screening tool)^h^ [27, 28]		O	O	O	O
J. Sleep	-Pittsburgh Sleep Quality Index (PSQI)^i^ [29]	O	O		O	O
K. Medical Use	-Medical center use, Unmet health needs [30]	O	O		O	O
	-Screening behavior, Insurance [4, 30]		O	O	O	O
L. Health Communication	-eHEALTH Literacy [31]		O	O	O	O
	-Health anxiety (Whiteley Index 7) [32]		O		O	O
	-Health information [33]	O	O	O	O	O
	-Health related Application use [33]	O	O			
	-Media use	O				
	-Communication Behavior [34]			O		
M. Social Support	- Multidimensional Scale of Perceived Social Support (MSPSS)^j^ [35]		O	O	O	O
N. Violence, Discrimination, Gender role	-Childhood violence exposure [5]		O	O	O	O
	-Discrimination (Gender, Work, Elderly) [30, 36]	O	O	O	O	O
	-Sexual harassment or violence [5]		O			
O. Gender role and Housework	-Gender role [5]	O	O	O	O	O
	-Housework share [37]		O	O	O	O
P. Socio-economic Status	-Education, Household Income, [4]		O	O	O	O
	-Employment status, Occupation, [4]		O	O	O	O
	-Satisfaction of money for basic living needs [30]		O	O	O	O
	-Family’s socio-economic status, Part time job, Study time [3]	O				
Q. Demographic Characteristics	-Marital Status [4],		O	O	O	O
	-House member, Housing type [4]		O	O	O	O
	-Previous work experience [37]		O	O	O	O
V. Sexual Life	-Contraception experience [30], Post-coital contraception use		O			

^a^ *PSS* Perceived Stress Scale, ^b^ *PHQ* Patient Health Questionnaire, ^c^ *EPDS* Edinburgh Postnatal Depression Scale, ^d^ *GDS* Geriatric Depression Scale, ^e^ *SF36* Medical Outcomes Study Questionnaire Short Form 36 Health Survey, ^f^ *PMS* Premenstrual syndrome, ^g^ *IPAQ* International Physical Activity Questionnaire, ^h^ *T-ACE* Tolerance Annoyance Cut down Eye opener, ^i^ *PSQI* Pittsburgh Sleep Quality Index, ^j^ *MSPSS* Multidimensional Scale of Perceived Social Support

A pilot study was conducted with 30 women per each life cycle stage (total of 150 women) to determine the feasibility and validity of the survey. Through the pilot study, we examined whether the questionnaire constructed by the experts was able to convey the correct meaning of each question to the general public. Based on the results from the pilot study, the wording of some of the expressions in the questionnaire was modified.

### Data collection

A total of 15,000 women aged 14–79 years completed surveys between April 2016 and June 2016. Trained interviewers from a professional research agency conducted door-to-door interviews to assess study eligibility. In order to select the survey subjects, the interviewers checked whether there was an eligible person in the household. Interviewers also visited schools and obstetrics and postnatal care centers to recruit eligible adolescent and pregnancy and post-partum women. The interviewers contacted subjects daily from 10 am until the evening, including weekends. Public holidays were excluded. Up to three attempts to contact an individual were made, at different times of the day. If contact was not made upon the third attempt, neighboring, alternative survey households were selected according to the predetermined contact order, and contact was tried again. Once the interviewers found eligible women, they explained the survey to and obtained signed consent from subjects who agreed to participate in the survey. Of the 37,334 people who were contacted, 15,084 interviews were completed. The survey response rate was 40.4%. Of these, a total of 15,000 interviews were included in the final analysis, excluding those who did not answer the main questions.

Respondent characteristics according to stage in life cycle are shown in Table 3. The mean age of the study participants was 41.2 years. Almost 80% of women resided in urban areas. Nearly 50% of women were included in the middle household income group (between $1700 and $3499). About 30% of women, not including adolescent girls, had a high school level of education. General characteristics differed according to stages in the female life cycle.

**Table 3:** General characteristics of the study participants in K-Stori

Variables		Adolescent girls N (%)	Childbearing women N (%)	Pregnant/Postpartum women N (%)	Menopausal women N (%)	Elderly women N (%)	Total N (%)
Total		3000 (100.0)	3000 (100.0)	3000 (100.0)	3000 (100.0)	3000 (100.0)	15,000 (100.0)
Age	Mean (SD)	15.5 (1.1)	31.2 (7.4)	32.9 (4.3)	54.4 (5.6)	71.8 (4.3)	41.2 (20.3)
Urbanization	Urban	2397 (79.9)	2400 (80)	2451 (81.7)	2394 (79.8)	2357 (78.6)	11,999 (80.0)
	Rural	602 (20.1)	600 (20)	549 (18.3)	606 (20.2)	643 (21.4)	3001 (20.0)
Monthly Household Income ($)	≤1699	412 (13.8)	190 (6.3)	49 (1.7)	313 (10.4)	1837 (61.2)	2801 (18.6)
	1700 ~ 3499	1466 (48.9)	1316 (43.9)	2046 (68.2)	1418 (47.3)	880 (29.3)	7126 (47.5)
	≥3500	1122 (37.4)	1494 (49.8)	905 (30.2)	1269 (42.3)	283 (9.4)	5073 (33.8)
Insurance Type	National Health Insurance (Self-employed)	-	750 (25.0)	534 (17.8)	1429 (47.6)	1684 (56.1)	4397 (36.6)
	National Health Insurance (Employed)	-	2238 (74.6)	2457 (81.9)	1539 (51.3)	1173 (39.1)	7407 (61.7)
	Medical Aid	-	6 (0.2)	7 (0.2)	30 (1.0)	139 (4.6)	182 (1.5)
	No insured	-	6 (0.2)	2 (0.1)	2 (0.1)	4 (0.1)	14 (0.1)
Marital Status	Single	-	1498 (49.9)	15 (0.5)	21 (0.7)	22 (0.7)	1556 (13.0)
	Married (with spouse)	-	1442 (48.1)	2940 (98.0)	2705 (90.2)	1896 (63.2)	8983 (74.9)
	Separated/Divorced	-	60 (2.0)	45 (1.5)	274 (9.1)	1082 (36.1)	1461 (12.2)
Education	No	-	3 (0.1)	0 (0.0)	14 (0.5)	428 (14.3)	445 (3.7)
	Primary School	-	5 (0.2)	0 (0.0)	181 (6.0)	1292 (43.1)	1478 (12.3)
	Middle School	-	17 (0.6)	12 (0.4)	400 (13.3)	815 (27.2)	1244 (10.4)
	High School	-	724 (24.1)	640 (21.3)	1703 (56.8)	426 (14.2)	3493 (29.1)
	University or Higher	-	2251 (75.0)	2348 (78.3)	702 (23.4)	39 (13.0)	5340 (44.5)

### Quality management

In order to ensure the reliability of the survey, step-by-step quality control was carried out in accordance with the survey preparation, survey process, and data entry and verification process. In order to enhance cooperation with the initial contact process, the purpose of and information about the K-Stori were posted on the homepage of The Korea Centers for Disease Control and Prevention, and National Cancer Center. To reduce non-sampling error, a standardized guideline was developed and disseminated to interviewers, and an education session was held for all interviewers prior to beginning the survey.

Regarding data entry and verification, a double check system was carried out by the researchers and assistant researchers to minimize errors when editing. Surveys were reviewed according to standardized guidelines upon completion of the entire survey. Inaccurate response items were confirmed by calling the subjects’ phone. Regional surveillance supervisors maintained a Computer Aided Telephone Interviewing system for 30% of randomly chosen surveys to check the accuracy of responses. In order to minimize errors in the coding process, experienced coding staff members were selected, and preliminary education on the coding guidelines was carried out. Punching error was minimized by operating a double punching system in which two people simultaneously punched the same questionnaire for all items.

### Ethical considerations

The study was approved by the institutional review board of the National Cancer Center, Korea (NCC2016–0062). An approved study description was provided to all eligible participants. The study description covered the research purpose, subject, content, duration, voluntary participation, withdrawal of consent, expected risks and benefits from participating in the research, and confidentiality. As the health information questions in this study assessed information on health behaviors, as well as mental and psychological factors, information security was strengthened. If the subjects agreed to participate in the study after reading the study description, participants were asked to provide written informed consent. For adolescents, because they were under 18 years of age, parental consent was obtained at the time of recruitment, or a parental consent form was sent to the adolescents’ homes and the survey was carried out after confirming consent. Women who were pregnant or who had recently given birth were allowed to consult with their spouse or partner and discuss their participation in the survey.

## Discussion

This study describes the rationale for and survey design of the K-Stori, which was undertaken to investigate health issues and unmet needs according to stages in the female life cycle among Korean women. The strength of this survey is that it utilizes questionnaire items designed to address the characteristics of women in different stages of the female life cycle. The questionnaire items were developed by obtaining the opinions of experts from multiple disciplines, conducting focus group interviews, and reviewing domestic and overseas survey tools. Currently, no study has evaluated health issues across the female life cycle with nationally representative data for Korea. The K-Stori will provide baseline data on the health problems faced by women in individual stages of their life cycle. The study will also serve as a basis for developing continuous surveillance platforms from which to identify health problems among Korean women. The data will highlight differences in health perceptions according to each life cycle stage and help identify differences in the health problems facing women as recognized by experts and those perceived by women themselves. Overall, the study will provides a basis for initiating women’s health projects and implementing policies targeting each stage of the female life cycle. Finally, this body of research will contribute to the literature on women’s health as a part of the continuous management and monitoring of women’s health and the securing of data and information with which to promote women’s health as stated in the Beijing Women’s Declaration.

## Abbreviations

EPDS: Edinburgh Postnatal Depression Scale
GDS: Geriatric Depression Scale
IPAQ: International Physical Activity Questionnaire
KCDC: Korea Center for Diseases Control and Prevention
K-Stori: Korean Study of Women’s Health Related Issues
MSPSS: Multidimensional Scale of Perceived Social Support
NCC: National Cancer Center
PHQ: Patient Health Questionnaire
PMS: Premenstrual syndrome
PSQI: Pittsburgh Sleep Quality Index
PSS: Perceived Stress Scale
TACE: Tolerance Annoyance Cut down Eye opener
